# Advances in machine learning for keratoconus diagnosis

**DOI:** 10.1007/s10792-025-03496-4

**Published:** 2025-03-30

**Authors:** Zahra J. Muhsin, Rami Qahwaji, Ibrahim Ghafir, Mo’ath AlShawabkeh, Muawyah Al Bdour, Saif AlRyalat, Majid Al-Taee

**Affiliations:** 1 Faculty of Engineering and Digital Technologies, University of Bradford, Bradford, BD7 1DP UK; 2 Department of Ophthalmology, The Hashemite University, Zarqa, Jordan; 3 School of Medicine, The University of Jordan, Amman, Jordan; 4 Liverpool, UK

**Keywords:** Detection, Early diagnosis, Severity staging, Corneal imaging modalities, Ophthalmology

## Abstract

**Purpose:**

To review studies reporting the role of Machine Learning (ML) techniques in the diagnosis of keratoconus (KC) over the past decade, shedding light on recent developments while also highlighting the existing gaps between academic research and practical implementation in clinical settings.

**Methods:**

The review process begins with a systematic search of primary digital libraries using relevant keywords. A rigorous set of inclusion and exclusion criteria is then applied, resulting in the identification of 62 articles for analysis. Key research questions are formulated to address advancements in ML for KC diagnosis, corneal imaging modalities, types of datasets utilised, and the spectrum of KC conditions investigated over the past decade. A significant gap between academic research and practical implementation in clinical settings is identified, forming the basis for actionable recommendations tailored for both ML developers and ophthalmologists. Additionally, a proposed roadmap model is presented to facilitate the integration of ML models into clinical practice, enhancing diagnostic accuracy and patient care.

**Results:**

The analysis revealed that the diagnosis of KC predominantly relies on supervised classifiers (97%), with Random Forest being the most used algorithm (27%), followed by Deep Learning including Convolution Neural Networks (16%), Feedforward and Feedback Neural Networks (12%), and Support Vector Machines (12%). Pentacam is identified as the leading corneal imaging modality (56%), and a substantial majority of studies (91%) utilize local datasets, primarily consisting of numerical corneal parameters (77%). The most studied KC conditions were non-KC (NKC) vs. clinical KC (CKC) (29%), NKC vs. Subclinical KC (SCKC) (24%), NKC vs. SCKC vs. CKC (20%), SCKC vs. CKC (7%). However, only 20% of studies focused on addressing KC severity stages, emphasizing the need for more research in this area. These findings highlight the current landscape of ML in KC diagnosis and uncover existing challenges, and suggest potential avenues for further research and development, with particular emphasis on the dominance of certain algorithms and imaging modalities.

**Conclusion:**

Key obstacles include the lack of consensus on an objective diagnostic standard for early KC detection and severity staging, limited multidisciplinary collaboration, and restricted access to public datasets. Further research is crucial to overcome these challenges and apply findings in clinical practice.

## Introduction

Keratoconus (KC) is an eye disorder where the cornea thins and bulges, causing irregular astigmatism and visual impairment [1]. It typically appears during early adolescence in both genders and progresses until the 40 s [2], affecting both eyes unevenly [3]. Despite decades of research, the precise cause of KC remains unclear. The onset and progression of KC are thought to result from a combination of genetic and environmental factors [4–6]. These factors likely interact in complex ways, contributing to the thinning and distortion of the cornea over time. 

The prevalence and frequency of KC vary significantly across different communities worldwide [3, 7, 8]. This variation is influenced by multiple factors, including differences in the populations studied, genetic predispositions, environmental influences, and the lack of standardized guidelines for defining and categorizing KC. In particular, the methods used to detect and classify the severity of KC can vary, leading to discrepancies in reported prevalence rates. Studies consistently indicate that the incidence of KC is notably higher in Middle Eastern and South Asian communities compared to other populations, suggesting a potential role of genetic and cultural factors unique to these regions [9].

Early diagnosis of KC is crucial for managing visual impairment and preventing progression. Treatment varies by disease stage and includes both surgical and non-surgical options. In the early stages, glasses and soft contact lenses correct nearsightedness, farsightedness, and astigmatism. As the disease progresses, rigid contact lenses help manage irregular astigmatism by allowing tear accumulation behind the lens, which restores the cornea’s shape [10]. While glasses and lenses can address refractive errors, they do not prevent disease progression. For progressive or at-risk cases, the standard treatment is corneal cross-linking.

In advanced stages, surgical options include corneal ring implants or corneal transplantation (keratoplasty). Severe cases may require partial-thickness or full-thickness (penetrating) keratoplasty to restore corneal function. Corneal collagen cross-linking (CXL) involves applying a riboflavin (vitamin B2) solution to the cornea as a photosensitizer, followed by exposure to ultraviolet A (UV-A) light at a wavelength of 370 nm [11]. This process promotes the formation of new collagen bonds, strengthening the cornea and preserving its smooth, spherical shape. Clinical trials have shown these effects can last up to seven years. [12]. Another surgical option is corneal ring implantation, where a C-shaped ring is placed in the corneal stroma to flatten the cornea and reduce astigmatism, improving visual acuity. In corneal transplant, also known keratoplasty, the patient’s damaged cornea is replaced by a donor’s cornea. However, many patients still require glasses or contact lenses for optimal vision afterward [13]. Along with the need for better refractive surgery planning and prevention of corneal ectasia, there has been an increased interest in the early and accurate diagnosis of KC.

Screening for suspect KC (also known as subclinical or SCKC) eyes is thought to be particularly important for early detection and monitoring of disease progression [14, 15]. Distinguishing healthy eyes (also referred to as normal or non-keratoconus [NKC] eyes) from clinically diagnosed keratoconus (CKC) is generally straightforward. However, identifying SCKC eyes presents a greater challenge, necessitating a more detailed assessment of corneal imaging studies. [16], including advanced 3D morphogeometric and volumetric analyses [17, 18]. It relies on findings from clinical examination, slit lamp evaluation, and corneal imaging modalities. As a result, there is a pressing need for the development of novel methods for the early detection of KC. The diagnosis of people with these conditions would enable early treatment to prevent or delay disease progression and the resultant visual impairment.

The application of Machine Learning (ML) as a diagnostic tool for a variety of medical diseases has been gaining momentum [19, 20]. Numerous supervised and unsupervised ML methods including deep learning have been developed to diagnose KC from its subclinical to more advanced stages [21, 22]. These methods aim to create software algorithms that can learn from data by minimising a loss function or increasing the likelihood [23].

In supervised learning, the model is trained on labelled data to detect keratoconus from new, unlabelled inputs. In unsupervised learning, the model identifies patterns in unlabelled data without explicit supervision. Deep learning [24], using multi-layer neural networks, is especially effective for segmenting and classifying large datasets of corneal images [25]. As automated screening tools, these ML methods can enhance diagnostic accuracy, improve KC diagnosis, and reduce the cost of new imaging hardware. They also lighten the workload of ophthalmologists [26], leading to better disease management and an improved quality of life for patients [27].

In order to ensure that ML is effectively incorporated into clinical practice for diagnosing KC, it is crucial to learn more about the opportunities, challenges and the applicability of the ML model in clinical settings. Consequently, this study aims to analyse the state-of-the-art findings on the feasibility and performance of ML methods in detecting KC. The primary objectives of this review are:

- Exploring recent advances in ML technologies for KC diagnosis, as well as providing details on the examined KC conditions, corneal imaging modalities, types and sizes of datasets used, and the performance levels attained by various methods.
- Identifying the ongoing challenges and constraints associated with the development of ML methods for KC diagnosis.
- Offering expert insights to tackle existing challenges while fostering multidisciplinary collaboration to seamlessly translate research findings into clinical practice.

The rest of this paper is structured as follows. The proposed research methodology is outlined in the next section. An overview of studies that have explored ML methods for KC diagnosis is presented in the following section. Subsequently, the results are presented and discussed, offering insights into the recent ML advances in KC diagnosis. Potential avenues for future research and development, tailored to both ML developers and ophthalmologists, are then proposed, along with a roadmap designed to facilitate the integration of ML models into clinical practice. Finally, the paper concludes by summarizing the key findings and recommending actionable steps to address the existing challenges and enhance the effectiveness of ML in KC diagnosis.

## Methods

This section provides a detailed overview of the libraries and resources utilized, as well as the search strategy employed to gather relevant data and literature. It also introduces the key research questions that form the foundation of this study, highlighting the critical areas of focus and the specific objectives the research aims to address.

### Libraries and search strategy

The primary digital libraries queried for this study include ACM Digital Library, IEEE Xplore, ISI Web of Science, BioMed Central, BMJ Best Practice, ProQuest, PubMed, ScienceDirect, Scopus, and SpringerLink. These libraries were systematically searched using a combination of carefully selected keywords, as follows:

- “keratoconus”
- AND (“artificial intelligence” OR “machine learning” OR “deep learning” OR “supervised” OR “unsupervised” OR “algorithm”)
- AND (“diagnosis” OR “detection” OR “examination” OR “analysis” OR “investigation” OR “severity” OR “grading” OR “interpretation”)

Initially, over 250 articles were collected. By evaluating their publication dates and relevance to the topic of interest, reviewing their abstracts, and removing unsuitable ones, a subset of 103 articles was identified as relevant. The selected articles cited in this review are then subjected to the following inclusion and exclusion criteria:

- The publication date must fall within the past 10 years.
- A research study is considered ineligible if it fails to meet one or more of the following criteria:
- Relevant to the subject addressed in this review article.
- A full-length original research publication excluding the review papers.
- Contains adequate details on the target KC condition(s), ML method(s), corneal imaging modality, dataset type and features, as well as information on the performance achieved in terms of at least one evaluation metric.
- Published in English language.

As a result of the thorough screening process, a total of 64 studies met the predefined inclusion criteria and were subsequently included in this review. These studies were selected based on their relevance to the research objectives, methodological rigor, and focus on ML applications in the detection and classification of KC.

### Research questions

The primary questions addressed in this study are:

**Q1**: What machine learning methods were employed, and how effective were they?

**Q2**: What kind of corneal imaging modalities and data types that were employed to detect KC?

**Q3**: What kind of datasets (images or numerical corneal parameters) were used, and were they publicly accessible or local?

**Q4**: What KC conditions were investigated?

## Machine learning in KC diagnosis

In the context of KC diagnosis, ML methods have been utilised to analyse a large set of data derived from corneal topography images [28–32], numerical corneal tomography data [33–40] or a combination of both, as well as other clinical and biometric measures [41].

The analysed studies reported a range of methods and classifiers, each demonstrating different performance levels in discriminating across several diagnostic targets: (i) non-keratoconus (NKC) versus clinical KC (CKC) eyes, (ii) NKC versus subclinical KC (SCKC) eyes, (iii) SCKC versus CKC (also known as frank KC), and (iv) various stages of disease progression. These studies were published in the context of academic research, and there are no adequate successful examples of ML methods being transformed to real-life applications. [42–44]. As a result, there is a standing need for developing an effective automated KC screening tools that can be successfully deployed into real-word application to address these challenges.

Tables 1 and 2 thoroughly examine the studies analysed in this review. Table 1 provides an overview of studies differentiating between NKC eyes, CKC eyes, SCKC eyes, or their combinations. Table 2 highlights studies focusing on distinguishing between various stages of KC severity. For studies employing multiple ML models, only the best-performing models are included.

**Table 1:** Summary of studies on ML methods for detecting NKC, SCKC, and CKC conditions

Study	Year	ML model Used	Imaging modality used	Dataset used			Input data type	Target conditions	Performance metrics (%)			
				Availability	Feature set	Subjects			Acc	Sen	Spe	Others
Smadja et al. [45]	2013	DT	GALILEI	Local	55	372	CP	NKC/CKC	–	100	99.5	–
								NKC/SCKC		93.6	97.2	–
Ramos-Lopez et al. [46]	2013	LR	Placido-based CSO Topography	Local	–	124	IM	NKC/ SCKC/CKC	–	33	78	–
Buhren et al. [47]	2014	DA	Orbscan IIz	Local	32	277	CP	NKC/SCKC	80.7	78.1	83.3	–
Saad et al. [48]	2014	DA	Placido topographer	Local	3	166	CP	NKC/CKC	–	94	100	AUC: 99.2
Chan et al. [49]	2015	DA	Orbscan IIz	Local	5	128	CP	NKC/SCKC	–	70.8	98.1	–
Lopes et al. [50]	2015	SVM	Pentacam	Local	6	560	CP	NKC/CKC	97.19	96	98	AUC: 991
Kovacs et al. [51]	2016	FFNN/FB NN	Pentacam	Local	15	135	CP	NKC/SCKC	–	90	90	–
Saad et al. [52]	2016	DA	OPD-scan	Local	8	176	CP	NKC/SCKC	–	63	82	–
Ruiz Hidalgo et al. [53]	2016	SVM	Pentacam	Local	22	860	CP	NKC/CKC	98.9	99.1	98.5	–
								NKC/SCKC	93.1	79.1	97.9	
Ruiz Hidalgo et al. [54]	2017	SVM	Pentacam	Local	25	131	CP	NKC/ SCKC/CKC	88.8	61	75	AUC: NKC/CKC: 99.78 NKC/SCKC: 93.55
Xu et al. [34]	2017	DA	Pentacam	Local	19	363	CP	NKC/SCKC	84.2	83.7	84.5	–
Ambrosio Jr et al. [55]	2017	RF + others	Pentacam + Corvis ST	Local	17	850	CP	NKC/SCKC	–	90.4	96	–
Francis et al. [56]	2017	LoR	Corvis ST	Local	10	458	CP	NKC/ SCKC/CKC	–	90	91	–
Lopes et al. [35]	2018	RF + others	Pentacam	Local	18	3168	CP	NKC/ SCKC/CKC	–	SCKC/CKC: 94.2	SCKC/CKC: 98.8	AUC: 99.2
										NKC/SCKC: 85.2	NKC/SCKC: 96.6	
Issaarti et al. [57]	2019	FFNN/FB NN	Pentacam	Local	5	851	CP	NKC/ SCKC/CKC	96.56	97.78	95.56	–
Chandapura et al. [58]	2019	RF	Pentacam + RCT Vue	Local	11	410	CP	SCKC/CKC	–	77.2	95.6	–
Xie et al. [59]	2020	DL/CNN	Pentacam	Local	–	6465	IM	NKC/ SCKC/CKC	94.7	97.8	99.2	AUC: 99.9
Kuo et al. [31]	2020	DL/CNN	Pentacam + Corvis ST + TMS-4	Local	–	354	IM	SCKC/CKC	–	28.5	97.2	–
Shi et al. [60]	2020	FFNN/FB NN + others	Pentacam + UHR-OCT	Local	49	121	CP	NKC/ SCKC/CKC	–	98.5	94.7	AUC: 93.0
Toprak et al. [17]	2020	LoR	MS-39	Local	15	202	CP	NKC/SCKC	–	96.8	94.5	–
Salomão et al. [61]	2020	RF	Pentacam + Corvis ST	Local	10	1295	CP	SCKC/CKC	–	100	–	AUC: 99
Zéboulon et al. [62]	2020	DL/CNN	Orbscan	Local	–	3000	IM	NKC	99.3 (overall)	100	99	–
								CKC		100	100	
								History of RS		98	100	
Bolarín et al. [63]	2020	LoR	Sirius Scheimpflug tomography + geometric modelling	Local	–	169	CP	NKC/CKC	69.8 (overall)	84.4	83.8	AUC: 84.3
Velázquez-Blázquez et al. [64]	2020	LoR	Sirius Scheimpflug tomography + geometric modelling	Local	27	178	CP	NKC/ SCKC/CKC	73 (overall)	NKC: 84	73	AUC: 87
										SCKC: 57	82	AUC: 69
										CKC: 63	97	AUC: 94
Shanthi et al. [38]	2021	SVM	Pentacam	Local	31	205	CP	NKC/ SCKC/CKC	CKC/NKC 91.8 CKC/SCKCK:100	CKC/NKC :94.2 CKC/SCKCK:100	CKC/NKC: 97.5 CKC/SCKC: 100	–
Herber et al. [40]	2021	RF	Pentacam + Corvis ST	Local	23	434	CP	NKC/SCKC	78	80	90	–
Castro-Luna et al. [36]	2021	RF	Pentacam	Local	81	81	CP	NKC/SCKC	89	SCKC: 86	SCKC: 93	–
Cao et al. [33]	2021	RF	Pentacam	Local	267	1692	CP	NKC/SCKC	98	97	98	–
Feng et al. [65]	2021	DL/CNN	Pentacam	Local	–	854	IM	SCKC/CKC	94.74	93.71	–	Pre: 94.1 F1: 93.89
Jiménez-García et al. [66]	2021	FFNN/FB NN	Pentacam + Galilei	Local	250	629	CP	NKC/CKC	–	70.8	80.6	–
Shetty et al. [67]	2021	RF	Pentacam	Local	31	366	CP	NKC/SCKC	91	89	81	AUC: 93 Pre: 90.4 F1: 90
Alazzam et al. [68]	2021	DL/CNN	Corvis ST	Local	–	1092	IM	NKC/SCKC	–	96.77	94.38	–
Yang et al. [69]	2021	DT	OCT	Local	4	174	CP	NKC/ SCKC/CKC	–	NKC: 100 CKC: 97.8 SCKC: 100	100	–
Al-Timemy et al. [70]	2021	DL/CNN	Pentacam	Public	–	4844	IM	NKC/ SCKC/CKC	97.7	98.0	–	AUC: 99.0
Song et al. [14]	2022	DT	BCT scan	Local	20	194	CP	NKC/SCKC	92.4	90.3	92.4	–
Almeida Jr et al. [71]	2022	DL/CNN	Pentacam	Local	50	2893	CP	NKC/ SCKC/CKC	–	86.02	83.97	AUC: 91.0
Cohen et al. [72]	2022	RF + others	Placido topographer	Local	94	4904	CP	NKC/CKC	91.5	94.7	89.8	AUC: 96.9
Ahn et al. [73]	2022	FFNN/FB NN	Pentacam	Local	n/a	1095	IM	NKC/CKC	–	96.4	–	–
Xu et al. [74]	2022	DA	Pentacam	Local	222	1108	CP	NKC/CKC	94.12	–	–	AUC: 98.3
Firat et al. [75]	2022	SVM	Pentacam	Local	9	682	CP	NKC/CKC	98.53	98.06	99.01	–
Gao et al. [76]	2022	FFNN/FB NN	Pentacam	Local	–	1040	IM	CKC/SCKC	–	CKC: 97.6 SCKC: 93.9	–	Pre: CKC: 95.1 SCKC: 96.1
Tan et al. [77]	2022	FFNN/FB NN	Corvis ST	Local	–	78	IM	NKC/CKC	98.7	97.4	100	Pre: 100
Lu et al. [78]	2022	RF	Pentacam	Local	12	622	CP	NKC/CKC	99	75	94.7	–
Kundu et al. [79]	2023	RF	OCT	Local	36	1125	CP	NKC/CKC	NKC: 95.6 CKC: 99.1	–	–	Pre: NKC: 95.1 CKC: 95.1
Dong et al. [80]	2023	LoR	Biomechanical	Local	–	632	IM	SCKC/CKC	N/A	97.8	99.2	AUC: 99.8
Kallel et al. [81]	2023	DL/CNN	Pentacam	Local	–	190	IM	NKC/CKC	99.74	98.71	–	Pre: 99.1
Civiero et al. [82]	2023	NB + other	ORA + Corvis ST	Local	–	154	IM	NKC/CKC	86	–	–	AUC: 94
Abdelmotaal et al. [83]	2023	DL/CNN	Pentacam, Corvis ST	Local	–	734	IM	NKC/CKC	89	82	96	AUC: 94 Pre: 95 F1: 88
Vinciguerra et al. [84]	2023	LoR	Corvis ST	Local	–	2473	CP	NKC/CKC	–	95.9	93.7	AUC: 0.99
Muhsin et al. [85]	2023	RF	Pentacam	Local	79	844	CP	NKC/CKC	100	100	100	Pre: 100 F1: 100
Muhsin et al. [86]	2024	RF + others	Pentacam	Local	79	1255	CP	NKC/ SCKC/CKC	99.60	99.01	–	Pre: 99.72 F1: 99.36 F2: 99.15

DA, discriminant analysis; DT, decision tree; KNNs, K-nearest neighbors; QDA, quadratic DA; SVM, support vector machine; RF, random forest; FFNN/FBNN, feedforward/feedback neural network; LR, linear regression; LoR, logistic regression; NB, naïve Bayes; DL/CNN, deep learning/convolutional NN

BCT, biomechanical computed tomography; ORA, ocular response analyzer; OCT, optical coherence tomography; Placido, Placido-based Topographers; IM, image (Topography, Tomography or Placido); CP, Corneal Parameters

Acc, accuracy; Sen, Sensitivity (or Recall); Spe, specificity; Pre, precision; AUC, area under the curve; F1, F1-score; F2, F2-score

NKC, non-KC; SCKC, subclinical KC; CKC, clinical KC

**Table 2:** Summary of studies on ML methods for KC severity staging

Study	Year	ML model used	Imaging modality used	Dataset used			Input Data type	performance metrics (%)			
				Availability	Feature set	Subjects used		Acc	Sen	Spe	Others
Yousefi et al. [87]	2018	Unsupervised Learning	CASIA SS-1000 (OCT)	Public	420	3156	CP	–	–	94.1	–
Lavric et al. [88]	2020	SVM + others	CASIA SS-1000 (OCT)	Public	8	3151	CP	94	89.5	96	–
Issarti et al. [89]	2020	FFNN/FBNN	Pentacam	Local	49	812	CP	97.6	85.2	70	–
Hallett et al. [29]	2020	Unsupervised Learning	Pentacam	Local	29	124	CP	80	–	–	AUC: 89
Cao et al. [37]	2020	RF + others	Pentacam + CASIA SS-1000 (OCT)	Local	11	88	CP	97	94	90	–
Aatila et al. [28]	2021	RF	CASIA SS-1000 (OCT)	Public	446	3162	CP	98	98	–	Pre: 98
Malyugin et al. [39]	2021	DA	Pentacam	Local	490	47,419	CP	97	–	–	AUC: 95
Lavric et al. [90]	2021	SVM + others (5-classes)	Pentacam	Local	38	5881	CP	–	–	–	AUC: 88
Kamiya et al. [91]	2021	DL/CNN	Placido topographer	Public	n/a	179	IM	87.78 (ave)	–	–	AUC: 93.68 (ave)
Shetty et al. [67]	2021	RF	Pentacam	Local	n/a	366	CP	90	–	97.7	Pre: 94.1
Priya et al. [16]	2022	SVM	CASIA SS-1000 (OCT)	Public	447	3164	CP	90	–	97.7	Pre: 94.1
Zorto et al. [92]	2023	DT + others	Pentacam	Local	5	237	CP	100	–	–	Pre: 100
Muhsin et al. [93]	2023	RF + others	Pentacam	Local	79	644	CP	98.62 (ave)	98.62	–	Pre: 98.70, F1: 98.66 F2: 98.64

RF, random forest; DL/CNN, deep learning/convolution neural network; SVM, support vector machine; FF/FB NN, feedforward/feedback neural networks; DA, discriminant analysis; DT, decision tree

OCT, optical coherence tomography; Placido, placido-based topographers; IM, image (Topography, Tomography or Placido); CP, corneal parameters

Acc, accuracy; Sen, sensitivity (or Recall); Spe, specificity; Pre, precision; AUC, area under the curve; F1, F1-score; F2, F2-score

## Results

A comprehensive review of the recent advances in ML methods for KC diagnosis is carried out to analyse 64 studies which were published within the past 10 years. The analysed studies are chronologically organised and summarised based on several criteria: the choice of ML method/classifier, corneal imaging modality, dataset accessibility (public or local), the number of features, the diagnosis target, and the achieved performance measured by specific metrics. Most of the examined studies (73.44%) were published between 2020 and 2024, while the remaining 26.56% were published between 2013 and 2019. This clearly indicates that the use of ML in KC diagnosis has become a growing topic of interest within the research community.

The research questions posed for this study are addressed based on the examined studies, as follows:

### Q1: What machine learning methods were employed, and how effective were they?

**Fig. 1:**
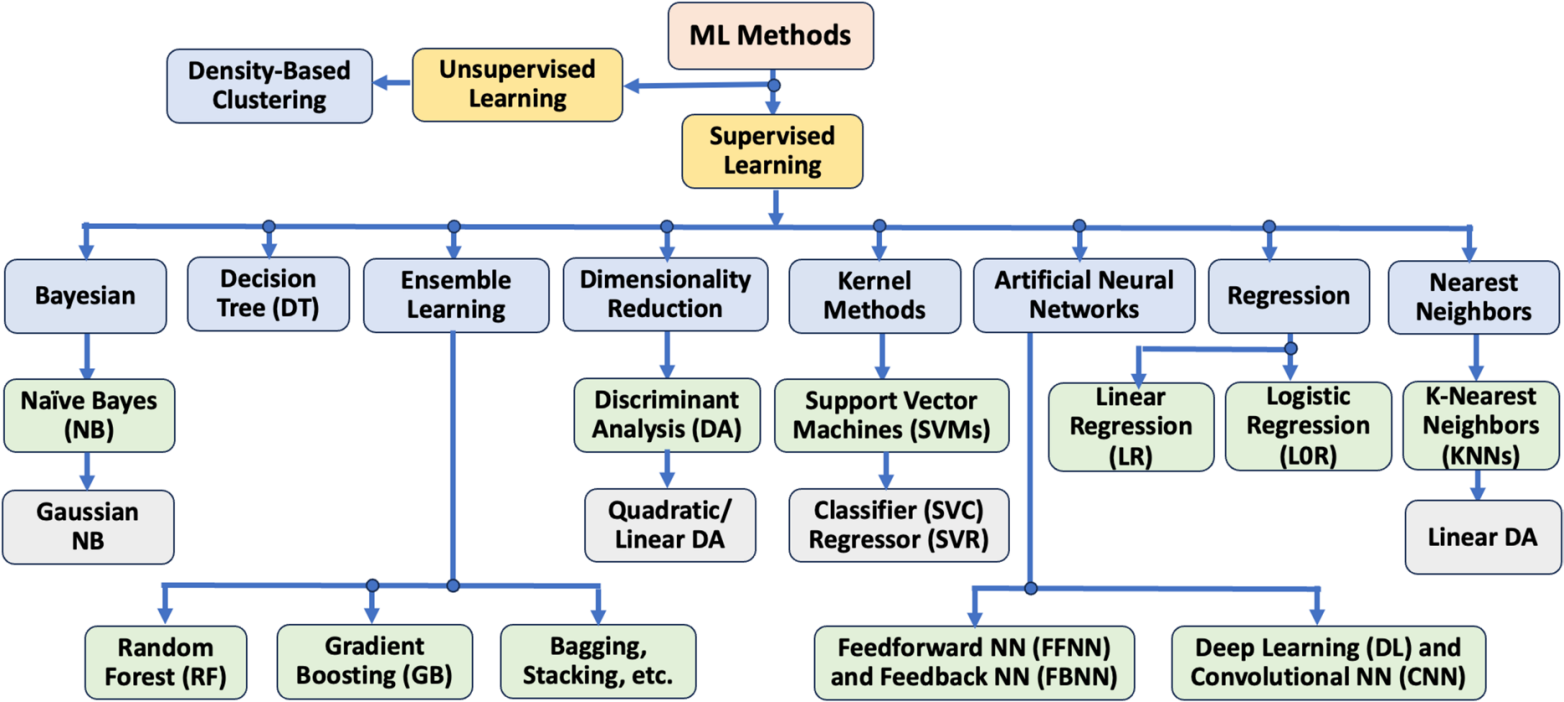
Base ML methods in KC diagnosis

Previous studies on ML algorithms for KC diagnosis employed a variety of supervised and unsupervised methods, as depicted in the hierarchical diagram in Fig. 1. The key principles behind these algorithms are summarized in Table 3. When implementing these techniques, careful selection of relevant parameter combinations from larger parameter sets, along with consideration of various demographic and clinical features associated with KC, was crucial for optimal performance.

**Table 3:** Key principles of ML algorithms illustrated in Fig. 1

Algorithm	Description
Decision trees (DTs)	DTs are versatile algorithms employed for both classification and regression tasks. It organizes data into a tree-like structure, where each internal node represents a decision point based on a specific feature, and each leaf node corresponds to a predicted outcome. The DT algorithm iteratively partitions the dataset into subsets by selecting feature values that provide the most effective prediction of the target variables (i.e., diagnoses labels)
Discriminant analysis (DA)	DA is a statistical technique for classifying data into predefined categories based on feature values, aiming to identify the best feature combination to separate classes. It is employed to predict categorical outcomes from the predictor variables. There are two primary types: (i) Linear Discriminant Analysis (LDA), which identifies a linear combination of features to distinguish between classes, and (ii) Quadratic Discriminant Analysis (QDA), which accommodates class-specific covariance matrices, enabling the use of a quadratic decision boundary for improved flexibility in classification
Feedforward/feedback neural networks (FFNNs/ FBNNs)	FFNNs and FBNNs are two neural network architectures with distinct characteristics. FFNNs are the simplest type, in which information flows in a single direction—from input to output—through hidden layer(s). In contrast, FBNNs enable information to persist through feedback connections, where the output of a neuron is looped back into the network as input for subsequent time steps, making them particularly well-suited for processing and analysing sequential data
Deep learning (DL) and convolution NN (CNN)	DL uses deep Artificial Neural Networks (ANNs) to model complex problems. These networks automatically learn hierarchical data representations, making them exceptionally well-suited for tasks such as image recognition, speech analysis, and natural language processing. DL models require large datasets and significant computational power, often leveraging GPUs (Graphics Processing Units)) for faster training. CNNs, a specialized type of DL model, are designed for grid-like data (e.g., images), automatically learning spatial feature hierarchies, which makes them highly effective for image-related tasks such as corneal topography images
Gradient boosting (GB)	GB is an ensemble learning algorithm used for both classification and regression tasks. It constructs a model incrementally by combining the predictions of multiple weak learners, typically decision trees, to progressively enhance accuracy and performance. Each tree tries to fix the residuals from the previous one, leading to a better performance. Popular variants of GB include XGBoost, LightGBM, and CatBoost, which are known for their efficiency and performance
K-nearest neighbors (KNN)	KNN is a simple algorithm employed for classification and regression tasks on small to medium-sized datasets. It operates by identifying the k nearest data points (neighbors) to a given input and making predictions based on the values of these closest points. Its simplicity comes with trade-offs like computational expense and sensitivity to noisy or high-dimensional data. However, with proper feature scaling and careful selection of k, KNN can be a powerful tool for pattern recognition and prediction tasks
Linear regression (LR)	LR is a fundamental ML algorithm that is simple, interpretable, and effective for many real-world applications. It establishes a relationship between a dependent variable (target) and one or more independent variables (features) by fitting a linear equation to the data, with the objective of predicting the target variable from the input features. While it struggles with non-linear relationships, extensions like regularization or polynomial regression can address a broader range of problems. Effective feature engineering and validation are key to optimizing its performance
Logistic regression (LoR)	LoR is an algorithm used for binary and multi-class classification tasks. Unlike LR, which predicts continuous outcomes, LoR predicts probabilities for discrete classes. The model outputs probabilities that are then converted into class labels using a certain threshold (e.g., 0.5). Its simplicity and ability to provide interpretable results make it a popular choice in many fields. However, its reliance on linearity and sensitivity to outliers mean it may underperform in complex scenarios compared to non-linear models
Naïve Bayes (NB)	NB is a group of probabilistic classifiers (based on Bayes’ Theorem), which predicts the class of a data point by estimating the probabilities of each class given the input features. The model is called "naïve" because it assumes conditional independence between features given the target class. Despite its simplicity, NB is highly effective for various classification tasks, especially with categorical data. Its speed, efficiency, and ability to adapt to various data types make it a valuable tool for many ML applications
Random forest (RF)	RF is a powerful ensemble learning algorithm commonly employed for classification and regression tasks. It constructs multiple decision trees during training and combines their predictions to enhance accuracy and robustness It works well on structured data, handling non-linear relationships, high-dimensional features, and noisy datasets. While it can be computationally expensive, its robustness and feature importance insights make it a go-to choice for many ML applications including its popularity in KC diagnosis
Support vector machines (SVMs)	SVMs are versatile algorithms employed for both classification (SVC) and regression (SVR) tasks. They share similar principles but have distinct objectives. SVMs excel in high-dimensional spaces and non-linear problems with the right kernel. They offer strong theoretical guarantees and can perform well on small, clean datasets. However, their computational complexity and sensitivity to hyperparameters make them less suitable for large or noisy datasets without careful tuning

**Fig. 2:**
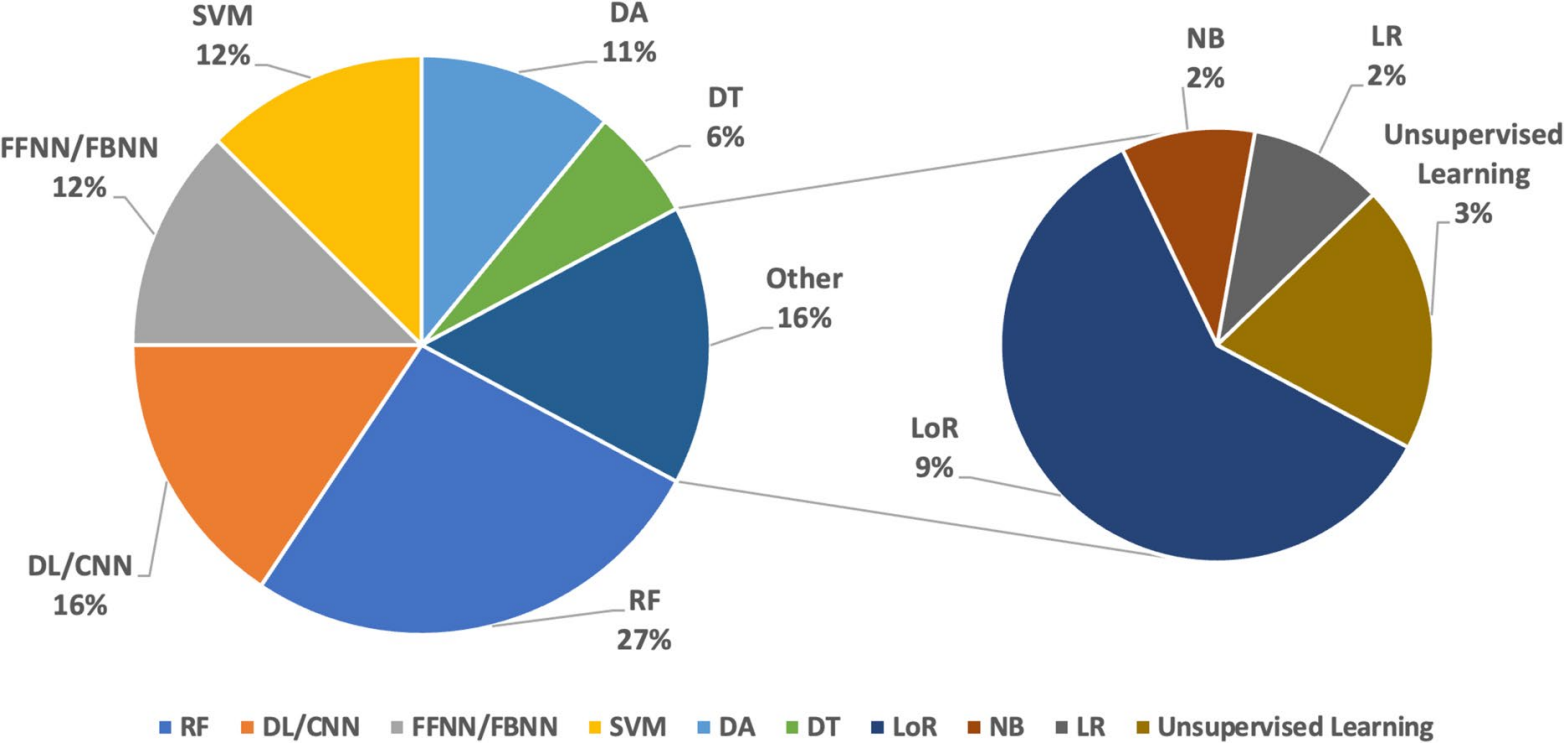
The utilisation distribution of the supervised/classifiers in KC diagnosis. RF, random forest; DL/CNN, deep learning/convolution neural network; SVM, support vector machine; FFNN/FBNN, feedforward/feedback neural networks; DA, discriminant analysis; DT, decision tree; LoR, logistic regression; NB, naïve Bayes

The analysed research showed that KC diagnosis was mostly based on supervised classifiers (97%); of these, the Random Forest (27%), followed by Feedforward and Feedback Neural Networks (12%), and Support Vector Machines (12%) were the most used algorithms for the corneal parameter-based classifiers. The Deep Learning including Convolution Neural Networks (16%) were also common in image-based classifiers as shown in Fig. 2. On the other hand, only few studies (3%) were utilised unsupervised methods. Overall, these classifiers exhibited varying performance levels in classifying different KC conditions, as shown in Table 1. Most of the studies utilised the accuracy, sensitivity (or recall), and specificity, as the criteria for measuring the performance of the developed models; in several investigations, the AUC (area under the curve), precision, F1 and F2 scores were also utilised as performance metrics. However, comparing the performance characteristics reported in the examined studies proved to be challenging for several reasons, including: (i) the lack of one diagnosis standard for KC diagnosis, (ii) unavailability of adequate publicly accessible datasets that can be used as a benchmark, and (iii) the utilisation of a range of imaging modalities and input parameters.

### Q2: What kind of corneal imaging modalities and data types that were employed to detect KC?

These has been a range of corneal imaging modalities that were employed to obtain different input data types including corneal parameters, images (Pentacam, Orbiscan, OCT, Corvis ST and others), corneal biomechanical properties, and others. he Pentacam, used in 56% of the reviewed studies, emerged as the preferred corneal imaging modality due to its detailed assessment of corneal shape, thickness, and curvature—key parameters for KC diagnosis.

### Q3: What kind of datasets (images or numerical corneal parameters) were used, and were they publicly accessible or local?

**Fig. 3:**
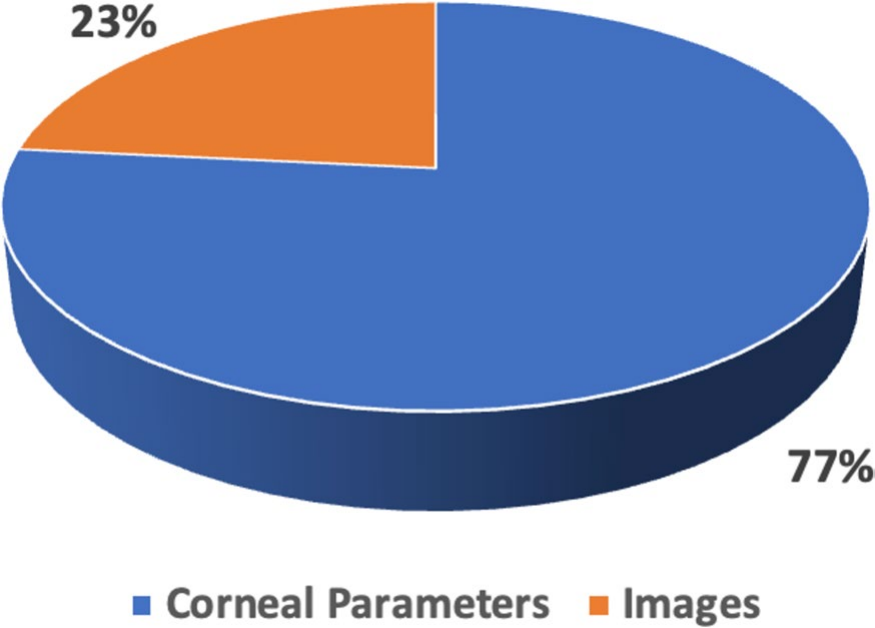
Comparison between input data types

As illustrated in Fig. 3, the analysis of the reviewed studies reveals that 77% of the research relied on numerical corneal parameter datasets, while the remaining 23% utilized image-based datasets. This distinction highlights a clear preference for numerical data in most KC detection/diagnosis studies. Furthermore, most of the examined studies (91%) utilized local datasets, suggesting that researchers are predominantly working with region-specific or institution-specific data that may limit the generalizability of the findings.

In contrast, only a small fraction of the studies (9%) made use of publicly available datasets. Notably, three public datasets were identified in the literature: two image datasets—one generated using a Placido disc-based system and reported in Kamiya [91], and another derived from a Pentacam imaging device, as cited in Al-Timemy [70]. Additionally, a corneal parameter dataset sourced from the CASIA SS-1000 (OCT) device was mentioned in [87].

**Fig. 4:**
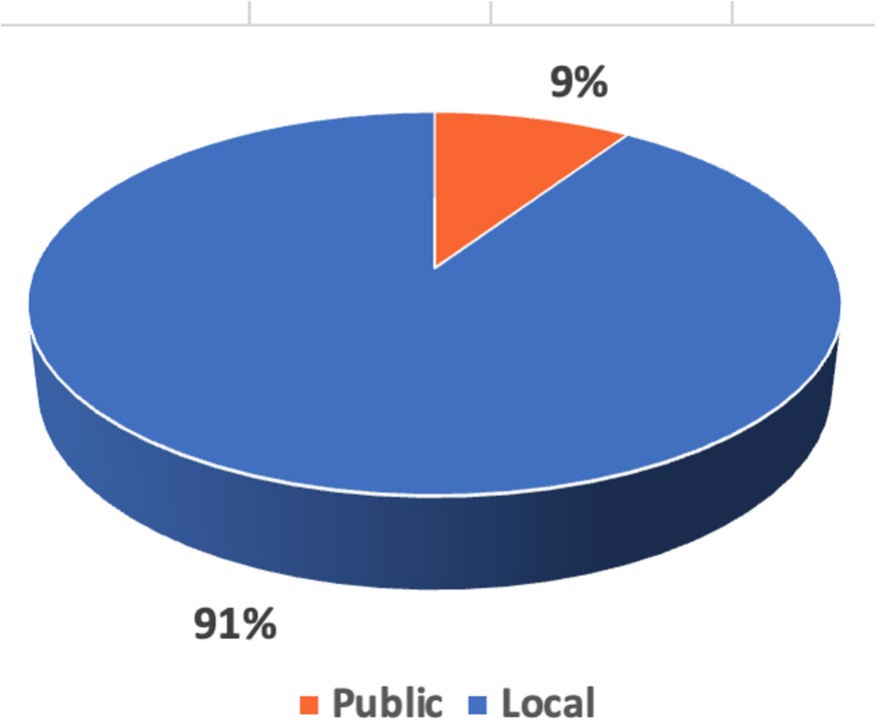
Comparison between local and public datasets utilisation

Despite being freely accessible, these public datasets have not been fully leveraged by the research community. As shown in Fig. 4, only 9% of the studies in the analysis incorporated these datasets, indicating a significant underutilization. This underuse may be attributed to various factors, including limited awareness of available resources, concerns about data quality, or the challenges in adapting public datasets to specific research needs. This trend suggests a potential area for growth in the field, where broader adoption of public datasets could enhance the diversity and robustness of machine learning models for KC diagnosis, potentially leading to more universally applicable diagnostic tools.

### Q4: What KC conditions were investigated?

**Fig. 5:**
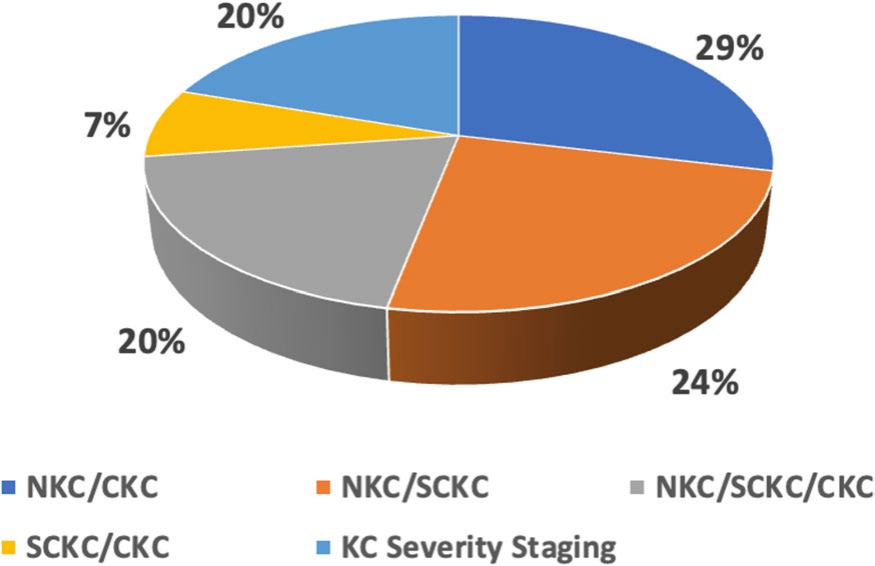
Distribution of studies based on the target KC conditions. NKC, non-KC; CKC, clinical KC; SCKC, subclinical KC

The reviewed research focused on five primary combinations of KC conditions: NKC vs. CKC, NKC vs. SCKC, NKC vs. SCKC vs. CKC, SCKC vs. CKC, and the classification of different stages of KC severity. As shown in Fig. 5, particular emphasis was placed on binary classifications between NKC and CKC (29%), followed by NKC and SCKC (24%). A further 20% of studies addressed the classification of NKC, SCKC, and CKC in a multi-class setting. However, only 20% of the studies have focused on the detection of various severity stages of KC, highlighting a need for further research in this crucial area. The smallest subset of studies (7%) was concentrated on differentiating between SCKC and CKC. Most of these studies were carried out within academic research settings, and none focused on detecting all KC conditions using a single practical application.

There is still some debate over the precise definition of SCKC, and there is no established standard for classifying an eye as having SCKC [88, 94, 95]. Additionally, in SCKC, the slit lamp-based examination is typically normal, which could fail to show any suspicious signs. The visual acuity and refraction, on the other hand, may not be significantly affected yet at this early stage. Hence, corneal tomography is the sole valid method for establishing a diagnosis [96]. As a result, the diagnosis of SCKC is accomplished subjectively using the judgment of expert ophthalmologists upon evaluation of examination findings [97].

## Discussion

As we anticipate the future, it urges us to explore avenues for fostering broader adoption of ML in KC diagnosis, all while considering the benefits for patients, healthcare providers, and the needs of developers. Our review focused on a set of key questions aimed at pinpointing the limitations and challenges contributing to the considerable gap between the development and implementation of ML algorithms in KC diagnosis. The limitations identified in the reviewed research are detailed below, accompanied by a proposed roadmap model for deploying ML models into clinical practice. Recommendations are also provided for both the scientific community and stakeholders, including ML developers and ophthalmologists. The aim is to foster collaboration and facilitate the creation of a more robust and comprehensive diagnostic tool for KC.

### Roadmap model for deploying ML models into clinical practice

**Fig. 6:**
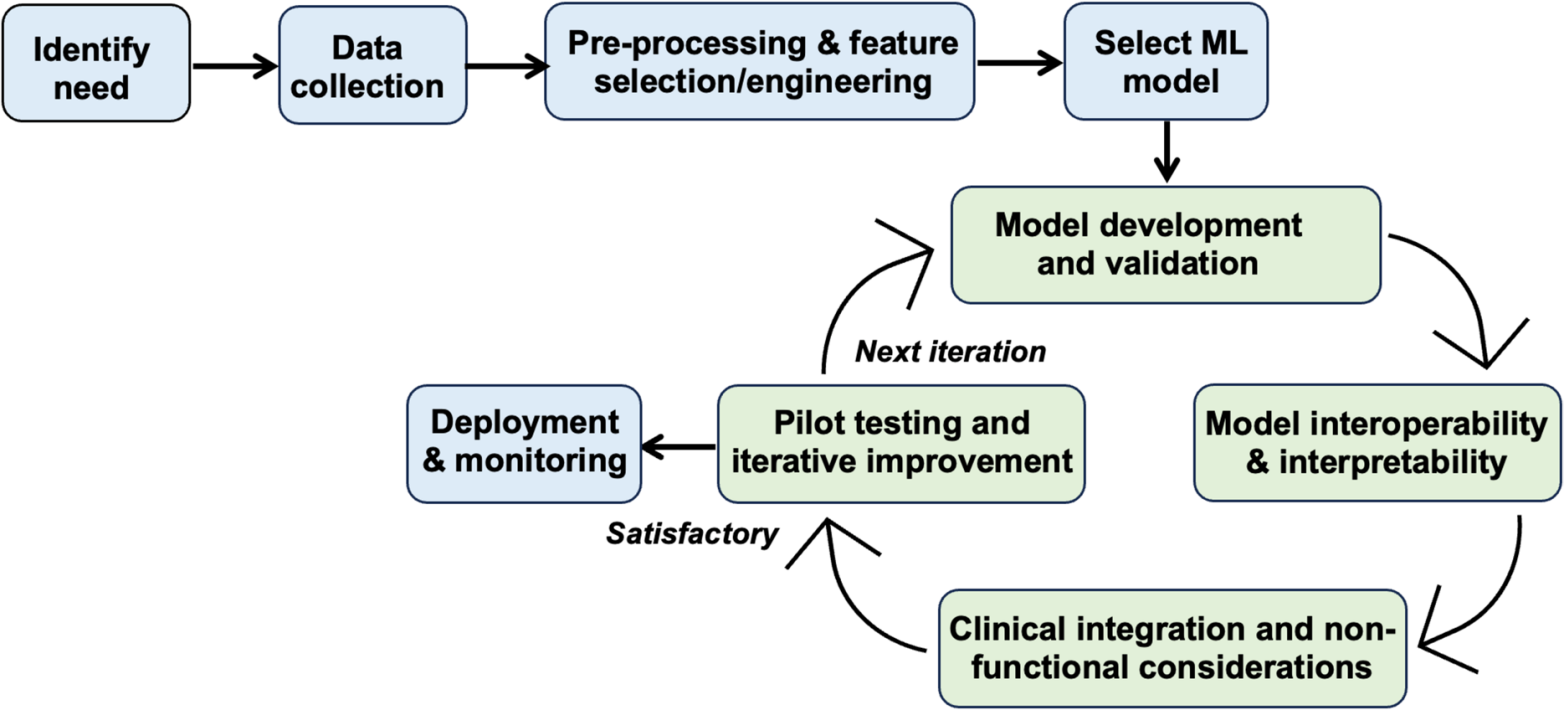
A roadmap model for the development and deployment of ML models into clinical practice

Deploying ML models into clinical practice requires careful consideration of various factors, including functional and non-functional consideration (e.g. model interpretability and interoperability, regulatory compliance and ethical considerations, user experience and others). A roadmap model outlining the key steps for developing and deploying ML models in clinical settings is suggested in Fig. 6. By following this model, healthcare organisations can successfully deploy ML models into clinical practice while addressing other challenges such as data bias and user acceptance.

To create a widely embraced ML model, the development approach needs to be centred around the user and undergo iterative refinement [98, 99]. Rather than developing a complete prototype upfront and seeking feedback afterward, each stage of prototype development should involve sharing the outcomes with end-users for their evaluation and feedback. This method commences with a profound comprehension of user needs, interface modalities, and interactions conducive to the ease of using the diagnostic tool for its intended users.

Achieving this objective entails soliciting feedback from the target users to understand the challenges they encounter in clinical practice and the improvements they anticipate from the ML model. Subsequently, the gathered data undergoes pre-processing and analysis utilising various statistical and visualisation techniques. This stage typically involves: (i) cleansing the data to address missing values, outliers, and inconsistencies. (ii) conducting feature selection and engineering to extract pertinent features from the raw data, and (iii) normalising or scaling the features as necessary to enhance model performance.

After pre-processing the collected data and identifying the effective features, an appropriate ML model is selected based on the nature of the problem (e.g., classification, regression). The data is then split into training, validation, and test sets. Next, the model is trained using the training data and its hyperparameters are optimised using the validation set. The model’s performance is then evaluated on the unseen test set and compared against clinical standards and expert opinions for validation. Assessing the model’s interoperability and interpretability is another essential consideration to ensure clinicians can understand and trust the model’s predictions in a clinically relevant manner.

The clinical integration and non-functional aspects involve assessment of the regulatory compliance and ethical considerations, including patient consent, fairness, and bias mitigation, throughout the deployment process. It also extends to the development of an intuitive user interface (UI) for clinicians to interact with the ML model seamlessly within their clinical workflow.

For example, it is important to ensure that the UI displays relevant patient information, model predictions, and confidence intervals in a clear and actionable manner. At the end of each development iteration, pilot testing of the model is conducted to gather feedback from clinicians and identify potential issues and this iterative cycle repeats as necessary until satisfactory results based on feedback and performance metrics, are achieved. The final stage in the suggested roadmap model involves deploying the ML model into clinical practice, ensuring smooth integration within the existing healthcare system. This stage also extends to:

- Monitoring model performance with regular updates, retraining, and clinical outcome evaluation.
- Training clinicians to interpret and integrate ML predictions into clinical decision-making.
- Promoting collaboration among data scientists, clinicians, and stakeholders for ongoing support and enhancement of the deployed model.

### Recommendations to ophthalmologists

1. It was reported in the global consensus document on diagnosis of KC [27] that "*abnormal posterior ectasia, abnormal corneal thickness distribution, and clinical non-inflammatory corneal thinning are mandatory findings to diagnose keratoconus.*" In the clinical practice, however, this definition is not straightforward since it did not specify thresholds or even parameters. To date, there is no objective method to detect KC, thus ophthalmologists must rely on their judgement and expertise, which can lead to subjective conclusions that are prone to human error. This problem is even more difficult in underdeveloped areas where ophthalmologists with specialised training are scarce.
2. There is a consensus that the early detection and monitoring of KC is vital for effectively managing symptoms associated with decreased visual acuity and astigmatism. This approach allows for timely treatment and helps prevent the progression of the disease. A delayed diagnosis of KC could lead to disease progression requiring a corneal transplant.
3. Currently in practice, ophthalmologists analyse corneal images and evaluate certain parameters, looking for patterns depending on the imaging device being utilised. However, the wide range of corneal imaging modalities, with parameters spanning from tens to hundreds, has made it challenging for general practitioners, optometrists, and other eye care professionals to interpret the data generated by these instruments. In this context, there is significant potential for ML to select a relatively small subset of the corneal parameters and develop effective models for KC screening, and severity staging which is not adequately explored in the literature
4. A universal set of parameters or thresholds for SCKC diagnosis [100] and distinguishing different stages of disease severity has yet to be established. This is likely due to the use of diverse diagnostic standards, imaging devices, and the absence of publicly available datasets that could serve as benchmarks for SCKC diagnosis and severity staging. As illustrated in Fig. 4, most of the analysed studies relied on local datasets. As a result, there is a critical need for publicly accessible datasets that combine multimodal data sources, including biomechanical, demographic, and genomic information.

### Recommendations to ML developers

1. The selection of a reliable subset of input features can improve the system performance by eliminating irrelevant variables, reducing the complexity of the data, and optimising algorithm prediction time [28]. This objective can be achieved by utilising a combination of effective feature selection methods and expert opinion.
2. The ML models explored in the studies focused on differentiating between several combinations of KC conditions: NKC vs. CKC, NKC vs. SCKC, NKC vs. SCKC vs. CKC, SCKC vs. CKC, as well as detecting various stages of KC severity. These conditions were addressed, employing individual base ML models, as depicted in Fig. 1. These foundational models lay the groundwork upon which more robust ensemble techniques can be explored. This strategy frequently yields enhanced performance and generalisation compared to individual base models. Despite their potential, ensemble learning techniques have been underreported in the literature for KC detection/diagnosis. Additionally, a more comprehensive KC diagnostic tool that address all KC conditions in a single application has not been reported yet in the literature. Achieving this objective entails refining ensemble learning methods for more resilient and accurate ML models towards developing a more comprehensive diagnostic tool for KC.
3. The field of ML in ophthalmology is one that is expanding quickly, and it is anticipated to play a significant part in the identification and diagnosis of KC. However, most of the reviewed studies on KC detection/diagnosis to date have been conducted in academic settings; it is unclear whether these studies have been translated into the real world [43, 44]. Therefore, more work is still needed to overcome this limitation by creating KC diagnostic tools that can be converted into practical applications that are accessible to a multiple eye-care clinics. Additionally, ML models deployed in real-world settings must not only exhibit high performance but also address challenges related to legality, ethics, data bias, trustworthiness, accessibility, user acceptance, and more. Combining recent developments in machine learning and web technologies can help achieve this goal by standardising diagnostic practices across multiple eyecare institutions on a national or regional level.
4. At the broader level, the scientific community and policy makers can concentrate on creating online platform-independent models that work with various corneal imaging instruments [57, 89], and conducting external model validation based on large patient populations. Additionally, there should be consensus on cut-offs parameters for SCKC and KC severity staging [89].

## Conclusion

Machine learning in ophthalmology, particularly for detecting KC, is advancing rapidly. Given the novelty and complexity of the subject, this paper provided the most comprehensive review available on this topic. It presented the latest and most relevant information in a concise format, enabling readers to gain valuable insights in a short amount of time.

This review revealed that most studies on ML applications for KC diagnosis and severity detection were conducted in academic settings, with limited translation into clinical practice. Notably, only 20% of these studies focused on KC severity stages, underscoring a need for further research in this critical area. Additionally, the reliance on base ML models highlights an untapped opportunity to leverage advanced ensemble learning techniques, which have the potential to provide more robust and accurate diagnostic outcomes.

A key finding of this review is the lack of a comprehensive diagnostic tool capable of addressing the full spectrum of KC conditions within a single application—an area that remains underexplored. Additionally, the absence of a consensus on objective diagnostic standards for KC, including subclinical forms and severity staging, presents a major challenge. The limited availability of publicly accessible datasets further complicates the ability to perform direct comparisons across studies, hindering progress in this field.

To overcome these challenges and enhance the utility of ML in KC diagnosis, the following actions are recommended:

1. Revising and expanding the decade-old global consensus document [27] to establish unified diagnostic criteria for subclinical KC diagnosis and severity detection. A standardized framework will facilitate consistency across studies and clinical implementations.
2. Creating open-access datasets for KC is a strategic investment in ophthalmic research. These datasets can address current data limitations and accelerate progress in understanding, diagnosing, and treating KC. This effort may involve generating large, balanced datasets using synthetic data that mimics real-world corneal imaging, enabling deep learning applications. Techniques like data augmentation (e.g., adjusting brightness, contrast, or angles), GANs (Generative Adversarial Networks) for realistic corneal scans, and VAEs (Variational Autoencoders) for controlled variations, such as different stages of corneal diseases can be utilized when real-world data are scarce or difficult to obtain.
3. Developing platform-independent ML diagnostic models compatible with a wide range of corneal imaging instruments is essential for broader applicability in diverse clinical environments. By developing models that integrate seamlessly with devices like slit lamps, OCT machines, and topographers, these tools can be accessible to healthcare providers across various settings. This flexibility promotes standardized care, improves diagnostic accuracy, and encourages collaboration across institutions, ultimately accelerating the adoption of ML-driven diagnostic tools in ophthalmology.
4. Fostering multidisciplinary collaboration through strengthening partnerships between clinicians, ML developers, healthcare institutions, and funding bodies. Ophthalmologists provide clinical expertise and ensure patient-cantered solutions, while ML developers bring technical innovations. Institutions play a pivotal role by facilitating access to diverse, high-quality data while ensuring compliance with ethical standards and patient privacy regulations. Finally, funding bodies are crucial for providing the financial resources needed to support the creation, maintenance, and expansion of public datasets. This form of multidisciplinary collaboration is vital for sparking innovation, bridging research gaps, and ultimately driving transformative advancements in KC research and treatment.

## Data Availability

No datasets were generated or analysed during the current study.
